# Singlet Exciton Fraction in Electroluminescence from Conjugated Polymer

**DOI:** 10.1038/s41598-017-02115-2

**Published:** 2017-06-06

**Authors:** Tzu-Hao Jen, Show-An Chen

**Affiliations:** Department of Chemical Engineering and Frontier Research Center on Fundamental and Applied Sciences of Matters, National Tsing-Hua University, Hsinchu, 30013 Taiwan, ROC

## Abstract

The efficiency of electrofluorescent polymer light-emitting diodes is determined by singlet exciton fraction (χ_S_) formation and its value still remains controversial. In this work, χ_S_ in spiropolyfluorene (SPF) is determined by analyzing transient emission of phosphor-dopant probe. The χ_S_ is found to range from 50% to 76%, depending on applied voltage. Higher applied voltage gives larger χ_S_. Besides, more rapid increment in χ_S_ with applied voltage is observed in the higher-molecular-weight polymer. The voltage or molecular weight dependence of χ_S_ suggests the probability of singlet exciton (SE) generation through triplet-triplet annihilation (TTA) is enhanced due to higher triplet exciton (TE) concentration at higher applied voltage or accommodation of more TEs in a polymer chain with high molecular weight, thereby increasing probability of TTA. At lower applied voltage, χ_S_ is contributed by charge recombination. Its value (χ_S_ ~50%) higher than the statistical limit 25% is in agreement with efficient interconversion between triplet and singlet polaron pairs (PP) and with larger formation rate of SE relative to that of TE.

## Introduction

In fluorescent organic light-emitting diodes (OLED) and polymer light-emitting diodes (PLED), only singlet exciton (SE) can undergo radiative decay. Triplet exciton (TE) is waste that limits its device efficiency. By doping with phosphorescent dopants containing noble metals like iridium and platinum (as guests) in organic molecules or polymers (as hosts), both SE and TE can be harvested and consequently internal quantum efficiency can be promoted toward 100%^1–3^. Metal-free organic phosphorescent materials are alternatives to organometallic phosphors for triplet harvesting^4, 5^. As compared to promoting intersystem crossing in organometallic phosphors, suppression of non-radiative relaxation processes from the triplet state to the singlet ground state in metal-free organic phosphorescent materials by making them a crystal or by embedding them into a matrix has been employed to enhance room temperature phosphorescence^4, 5^. However, a roll-off in efficiency at high current densities^6–8^ and slower response time of electrophosphorescent device may limit its application. Therefore, development of highly efficient electrofluorescent device is highly desirable, in which singlet exciton fraction (χ_S_) is one of key parameters that determines overall device efficiency

From simple quantum statistical consideration, charge recombination of electron and hole results in one SE and three TEs as was confirmed for OLED^9, 10^. However, the reported χ_S_ through charge recombination in conjugated polymers still have large differences ranging from 17% to 83%^10–19^. Most of these earlier measurements for PLED suffer from uncertainty including how to obtain accurate value of light out-coupling efficiency (η_C_)^12, 15^ or estimate possible difference in η_C_ between photoluminescence (PL) and electroluminescence (EL) in the relative methods involving ratio of PL and EL intensities for non-cancelable η_C_ in the estimation of χ_S_^10, 11, 13, 17, 19^. The electric-field-assisted pump-probe measurement could be one approach in determining χ_S_ without considering η_C_^16^. However, very high negative electric field (for example, the applied 1.7 MV/cm is corresponding to 17 V with the film thickness 100 nm) applied to the device can lead to carriers injection and affect the dynamics of excited state species. Besides, some studies adopted poly(p-phenylene vinylene) (PPV) derivatives with alkoxy side chains as investigated system^10–12, 15, 18^, which tends to form aggregates (the emitting species which is due to intermolecularly mutual interaction between two or more lumophores in the ground state by extending the delocalization of π-electrons over these conjugated segments)^20^. These aggregates can act as energy harvesters^20^ or recombination centers^21^ and thus will affect χ_S_ value obtained.

The extra SE can also be generated via collision between two TEs. This process is called triplet-triplet annihilation (TTA), and its occurrence has been supported by some experimental evidences^22–30^. For example, it has been reported that TTA can contribute to 20% increase in the external quantum efficiency (EQE) of a blue-emitting PLED^27^. Furthermore, the blue fluorescent PLED with 7.28% EQE have also been demonstrated by us ref. 31, exceeding 5% theoretical limitation assuming a η_C_ of 20%. Therefore, TTA can be regarded as an alternative way of recovering non-emissive TE as compared to utilizing the phosphorescent dopants.

Thermally activated delayed fluorescence (TADF), an up-conversion process from non-radiative TE to radiative SE via reverse intersystem crossing (RISC), has also been proposed to give extra SE and thus increase the efficiency of fluorescent device^32–35^. The green OLED with very high EQE 25.7% can be achieved via this approach^34^, which is comparable to that achieved in highly efficient phosphorescent OLED^2^. However, careful design of molecules with a small energy gap (ΔE_ST_) between singlet state and triplet state (ΔE_ST_ ≦ 0.2 eV)^36^ should be fulfilled for obtaining highly luminescent TADF materials.

Here, χ_S_ in the spiropolyfluorene (SPF) based PLED is evaluated by transient electroluminescence (TREL) technique. With the aid of addition of red-emitting phosphorescent dopant bis(2-benzo[b]thiophen-2-yl-pyridine) (acetylacetonate) iridium(III) (Ir(btp)_2_acac) as a TE probe in SPF and analyzing its transient emission, together with the predetermined energy transfer (ET) efficiency from SPF to Ir(btp)_2_acac and careful correction of enhanced intersystem crossing (ISC) rate of polymer host induced by phosphor dopant (external heavy atom effect)^37, 38^, χ_S_ in the SPF can be estimated accordingly. We find that χ_S_ in the SPF based PLED depends on the applied voltage and always exceeds the statistical limit (25%), ranging between 50% and 76%. Higher applied voltage leads to larger χ_S_. At low applied voltage, χ_S_ is contributed by charge recombination. Its value (χ_S_ ~50%) is higher than the statistical limit 25%. The electric field dependence of χ_S_ is suggestive of increasing SE generation through TTA since higher TE concentration at higher applied voltage can promote the chance of collision between them. Besides, more rapid increment in χ_S_ with applied electric field in SPF with higher molecular weight (SPF_HMW_, Mw = 210275 Dalton, polydispersity (PDI) = 1.34) than that in SPF with lower molecular weight (SPF_LMW_, Mw = 183509 Dalton, PDI = 1.66) was also observed, suggesting a generation of SE through TTA since a longer-chain SPF can accommodate more TEs in comparison with that in a shorter-chain SPF, thereby increasing the probability of intramolecular TTA. This result indicates that molecular weight of conjugated polymer can affect χ_S_ in PLED even though the molecular weight of SPF_HMW_ is higher than that of SPF_LMW_ only by a factor of about 1.15, which doesn’t exist in the OLED. In other words, we can manipulate χ_S_ possibly via chain orientation of conjugated polymer. These are the subtle points for development of electrofluorescent PLED with high efficiency.

## Results and Discussion

### Photophysical properties

**Figure 1:**
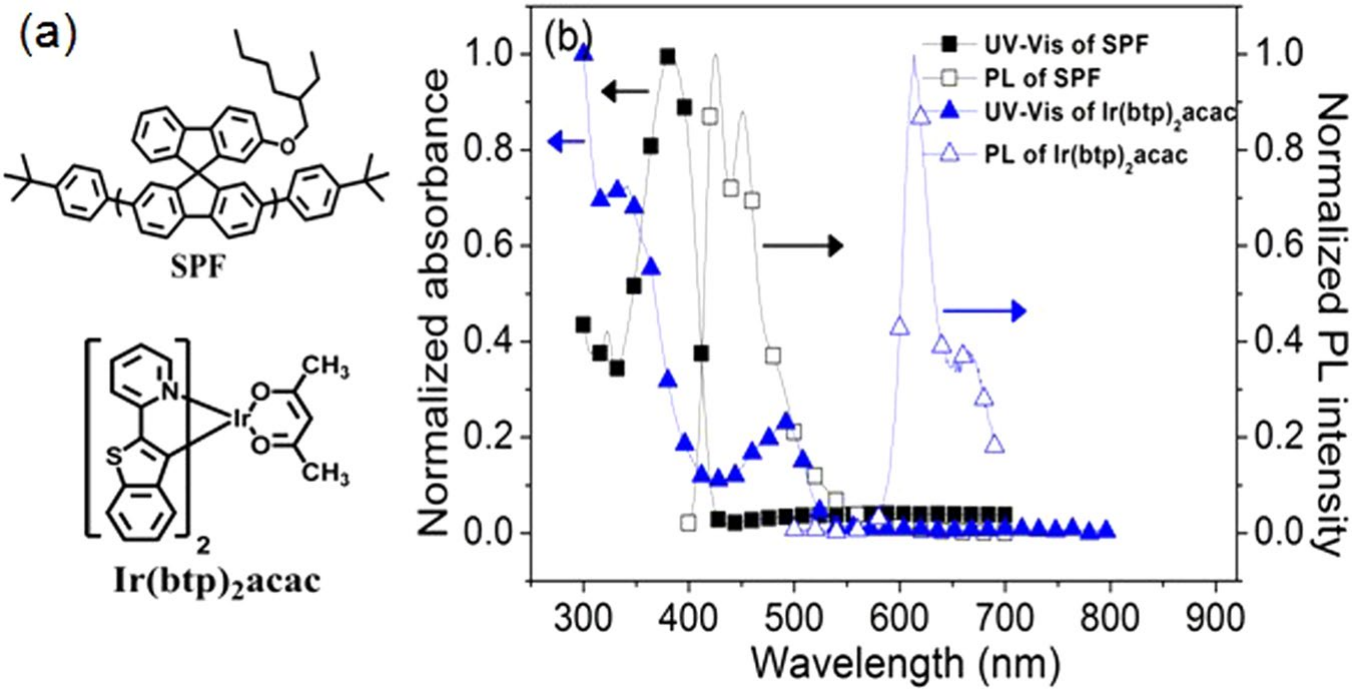
(**a**) Chemical structures of SPF and Ir(btp)_2_acac. (**b**) UV-Vis absorption and PL spectrum of SPF as thin solid film and those of Ir(btp)_2_acac in tetrahydrofuran solvent.

The SE and TE generated in the blue-emitting polymer SPF were probed by the red-emitting phosphorescent dopant, (Ir(btp)_2_acac). Their chemical structures and corresponding UV-Vis absorption and PL spectra are shown in Fig. 1. The singlet-singlet ET from host to guest is expected to occur due to the good spectral overlap between the absorption of metal-to-ligand charge-transfer band (MLCT^1^, 410–535 nm) of Ir(btp)_2_acac^39^ and emission spectrum of SPF. Besides, the triplet energies (E_T_s) of SPF and Ir(btp)_2_acac are 2.1 eV^40^ and 2.0 eV^41^, respectively, therefore, the exothermic triplet-triplet ET from SPF to Ir(btp)_2_acac is also expected. These results indicate that Ir(btp)_2_acac can act as an effective energy (SE and TE) harvester for the SPF. The model compounds normally used in photophysical studies, such as poly(2-methoxy-5-(2-ethylhexyloxy)-1,4-phenyle-nevinylene) (MEH-PPV) or polyfluorene (PFO), were not adopted here for the following reasons. For the orange-emitting MEH-PPV, its E_T_ is too low (1.3 eV)^42^ to find an appropriate phosphor with lower E_T_ so that exothermic triplet-triplet ET from MEH-PPV triplet takes place. Besides, MEH-PPV tends to form aggregates^20, 21^. These aggregates can act as energy harvesters^20^ or recombination centers^21^, which will affect χ_S_ obtained. The blue-emitting PFO with E_T_ of 2.18 eV^43^ could be a candidate for χ_S_ measurement using Ir(btp)_2_acac as a probe for TE, but the measurement may become complicated due to appearance of unexpected green emission (possibly from the “keto defect emission”^44^, “aggregates”^45^ or “excimer formation”^46^) or blue emission (possibly from a presence of β phase^47^). For SPF, its spiro structure can reduce the interchain interaction that leads to a broadening of the emission spectrum^48^. Besides, good emission color stability was reported for spiro-based blue-emitting polymers due to free from keto defect emission^49^.

### Time-resolved electroluminescence (TREL)

As mentioned above, χ_S_ in the SPF based PLED will be estimated with the aid of addition of emissive Ir(btp)_2_acac as TE probe to conjugated polymer. In fact, employing emissive phosphor as TE probe has been proposed for a determination of χ_S_ in OLED^9^, in which singlet-singlet and triplet-triplet ET efficiencies (ηET^S^ and ηET^T^) between host and guest along with EL and PL efficiencies of OLED with and without phosphor were measured to estimate χ_S_. The utilization of device EQE ratios of EL to PL in both phosphor doped and undoped devices in their theoretical development allowed them to cancel out η_C_. However, it is possible that η_C_ of PL and EL are not equal^44^ so that this simplification is not applicable and consequently χ_S_ value will be affected.

Here, a relative simple method based on a measurement of transient EL of phosphor dopant was employed to determine χ_S_ in the SPF based PLED. The observation of transient EL of phosphor dopant was originally proposed to give a direct evidence of SE and TE generated in the host harvested by the phosphor^50^. Accordingly, transient response of phosphor emission can be described by the following equation (detailed derivations are shown in the Supplementary Information (SI)):

1 $${[T]}_{G}=A\cdot \exp (\frac{-t}{{\tau }_{T}^{G}})+B\cdot [\exp (\frac{-t}{{\tau }_{T}^{G}})-\exp (\frac{-t}{{\tau }_{T}^{Hd}})]$$

where [T]_G_ is time dependent TE concentration in the phosphor and should be proportional to observed transient phosphorescence intensity of phosphor; τ_T_^Hd^ and τ_T_^G^ are TE lifetimes of host and phosphor guest, respectively. A is a parameter related to generation of phosphor TE by singlet-singlet ET; and B is that by triplet-triplet ET. Based on Equation 1, it has been shown that 40% more phosphor (platinum octaethylporphyrin, PtOEP) emission in electrically excited device compared to that in optically excited film with poly[4-(N-4-vinylbenzyloxyethyl,N-methylamino)-N-(2,5-ditert-butylphen-ylnapthamide)] (PNP), a non-conjugated polymer with conjugated moiety grafted onto the non-conjugated polymer backbone, as a host, suggesting that both SE and TE can be harvested by phosphor dopant^50^. Since A is proportional to SE concentration generated in the host and η_ET_^S^, and B is dependent on TE concentration generated in the host and η_ET_^T^, therefore, the ratio of SE to TE ([S]/[T]) can be expressed in terms of A, B, η_ET_^S^ and η_ET_^T^ as follows (see SI for detailed derivations):

2 $$\frac{[S]}{[T]}=(\frac{A}{C})\cdot (\frac{{\eta }_{ET}^{T}}{{\eta }_{ET}^{S}})\quad {\rm{and}}\quad {\chi }_{S}=\frac{[S]}{[S]+[T]}$$

C can be expressed in terms of B, τ_T_^Hd^ and τ_T_^G^ by the equation below:

3 $$C=B\cdot (1-\frac{{\tau }_{T}^{Hd}}{{\tau }_{T}^{G}})$$

The ET efficiency can be calculated from change of exciton lifetime upon phosphor doping^51^. These lifetimes can be obtained by time-resolved photoluminescence (TRPL) (for SE) and photo-induced absorption (PIA) (for TE), respectively. Therefore, we can determine [S]/[T] or χ_S_ from measurable parameters. Since both A and C (or B) are proportional to η_C_ of phosphor EL, that term can be cancelled out in the determination of χ_S_ (Equation 2). Note that measurements of TRPL and PIA should be carried out in the same device structure as for TREL to prevent possible difference in the decay process for exciton. Furthermore, the following assumptions are made in derivations. First, the ISC efficiency of phosphor guest is close to 100% (Φ_ISC_^G^ ~ 100%). Second, direct charge trapping on phosphor can be neglected. Finally, it is assumed that singlet-singlet ET is instantaneous relative to the timescale of the ongoing triplet-triplet ET. The validities of these assumptions are met in the present system (SPF and Ir(btp)_2_acac) and are justified below.

**Figure 2:**
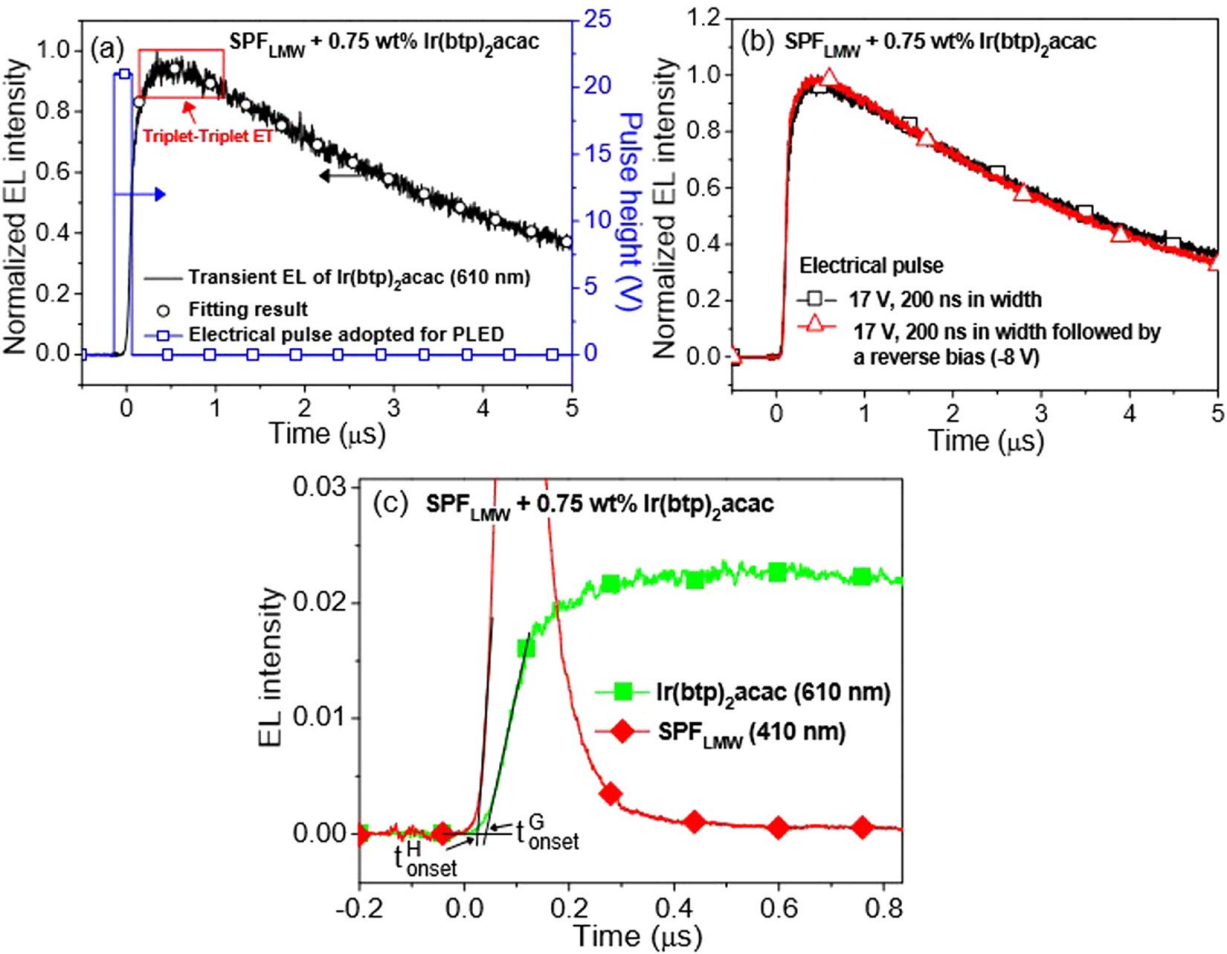
(**a**) The transient response of Ir(btp)_2_acac emission (610 nm) in 0.75 wt% Ir(btp)_2_acac doped SPF_LMW_. The rise after electric pulse indicates triplet-triplet ET from SPF to Ir(btp)_2_acac. The representative excitation condition (21 V, 200 ns in width) adopted for the PLED is also shown in the figure. The open circle shows fit to Equation 1. (**b**) The negligible effect of reverse bias applied on transient EL emission of Ir(btp)_2_acac (triangle) supports triplet-triplet ET from SPF to Ir(btp)_2_acac. (**c**) The onset times compared for SPF_LMW_ (410 nm, t^H^_onset_) and Ir(btp)_2_acac (610 nm, t^G^_onset_) emissions. The device structure is ITO/PEDOT:PSS/emitting layer/TPBI/Ca/Al. The representative excitation condition adopted for the PLED is 22 V (200 ns in width).

The TREL of Ir(btp)_2_acac at 610 nm in Ir(btp)_2_acac doped-SPF_LMW_ is shown in Fig. 2a (TREL, TRPL and PIA of SPF_HMW_ related system are provided in the SI). The device structure is ITO/poly(3,4-ethylenedioxythiophene):poly(styrenesulfonate) (PEDOT:PSS)/emitting layer/1,3,5-tris(2-henylbenzimidazolyl)benzene(TPBI)/Ca/Al, in which TPBI is the hole/exciton blocking layer. The utilization of TPBI can provide increased residence time of SPF TE in the luminescent region and thus enhance η_ET_^T^ ^6^. A rise (~several hundreds ns) after short electrical excitation pulse (200 ns) was observed (Fig. 2a), which can be ascribed to the triplet-triplet ET from the SPF to Ir(btp)_2_acac owing to its slower time scale as compared to that of singlet-singlet ET (it is about several tens ps as to be shown later) in the present system.

One may argue that this rise could be resulted from a recombination of remaining charge carriers trapped on the phosphor after electrical excitation with the carriers of the opposite type they captured (charge trapping) rather than triplet-triplet ET^52^. If phosphorescence rise is due to charge trapping, the additional negative bias applied after positive electrical excitation can empty traps by sweeping out the remaining charges in the emitting layer, leading to non-observed rise component in phosphor emission^50, 52^. However, Fig. 2b shows that transient phosphorescence was not affected by the reverse bias applied, suggesting that triplet-triplet ET rather than charge trapping is dominant in the present system.

To further support the above argument, the onset times of SPF_LMW_ (at 410 nm) and Ir(btp)_2_acac (at 610 nm) emissions under electrical pulse excitation were compared. The onset time is determined by extrapolating solid line from the background and from the straight onset of transient emission. If charge trapping exists in this blend system, the onset time of phosphor emission should be ahead of the SPF emission because electron and hole first recombine at phosphor dopant. However, the onset time of Ir(btp)_2_acac emission is delayed about 17 ns as compared to that in SPF (Fig. 2c), showing that charge trapping effect can be neglected in the present system.

From the above discussions, charge trapping effect can be safely neglected in the present system. Besides, the timescale of the singlet-singlet ET (it is about several tens ps as to be shown later) is much faster than that of triplet-triplet ET (about several hundreds ns). Along with nearly 100% ISC efficiency of the Ir(btp)_2_acac due to its strong spin-orbital coupling induced by the incorporated heavy atom (iridium)^53^, the assumptions in the present theoretical development can be totally satisfied. The open circle profile in Fig. 2a shows the fit to Equation 1, supporting transient response of Ir(btp)_2_acac emission can be well described by the equation developed. In other words, we can use developed equations to determine [S]/[T] of SPF. By fitting transient response of Ir(btp)_2_acac emission (Fig. 2a) with Equation 1, the lifetime of Ir(btp)_2_acac is about 5.5 μs, which is very close to the reported lifetime of Ir(btp)_2_acac in solution (5.8 μs)^41^. Therefore, the homogeneous dispersion of phosphor dopant within the film can be expected since aggregation of phosphor dopant will lead to significant reduction in phosphor lifetime due to effect of concentration quenching^54^. Besides, the dopant concentration of Ir(btp)_2_acac is only 0.75 wt%, the aggregation of phosphor dopant is also unlikely. Furthermore, although variation of local phosphor concentration is not considered in our theoretical model, the transient response of Ir(btp)_2_acac emission can be well described by the equation developed. From the above discussions, the influence of variation of local phosphor concentration on χ_S_ value can be safely neglected.

### The determination of η_ET_^S^

**Figure 3:**
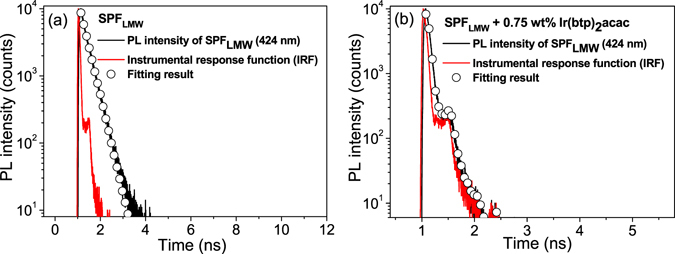
(**a**) TRPL of SPF_LMW_ at 424 nm for optical excitation at 387 nm, that of Ir(btp)_2_acac doped SPF_LMW_ is provided in the (**b**). The open circles are single exponential fits and average fall times (over three samples) of 293 ps and 36 ps can be obtained for pure and Ir(btp)_2_acac doped SPF_LMW_, respectively. These TRPL measurements were carried out in device geometry with the structure: ITO/PEDOT:PSS/emitting layer/TPBI/Ca/Al.

Figure 3 shows the TRPL of SPF_LMW_ at 424 nm (at the PL maxima of SPF), that in the presence of Ir(btp)_2_acac is also provided. They were both measured in the device structure for the reason mentioned previously. The fluorescence decay of SPF can be characterized by the single-exponential function and the resulting singlet lifetimes for SPF_LMW_ with and without Ir(btp)_2_acac (τ_S_^Hd^ and τ_S_^H0^) are 36 ps and 293 ps (averaged over three samples), respectively. The η_ET_^S^ then can be expressed in terms of τ_S_^Hd^ and τ_S_^H0^ by the expression η_ET_^S^ = 1 − (τ_S_^Hd^/τ_S_^H0^) (which is the ratio of ET rate to the total decay rate of polymer SE)^51^ and is calculated to be 88% in SPF_LMW_/Ir(btp)_2_acac blend system.

The above calculation of η_ET_^S^ is based on reduction of SE lifetime of SPF upon phosphor doping, the shorter the SE lifetime of SPF after phosphor doping is, the larger the η_ET_^S^ is. However, apart from singlet-singlet ET, the reduction of SE lifetime in phosphor doped polymer host can also be resulted from more efficient conversion of SE to TE due to increased ISC rate by external heavy atom effect^37^, resulting in overestimation of ET efficiency. Therefore, some corrections should be made. Based on TE induced absorption for pure and phosphor doped SPF, the corrected singlet-singlet ET efficiency (η_ET,C_^S^) and corresponding timescale for SPF_LMW_/Ir(btp)_2_acac are 76% and 47 ps. The timescale of singlet-singlet ET is much faster than that of phosphorescence rise (several hundreds ns) shown in Fig. 2a, supporting the rise component in phosphor transient emission is due to triplet-triplet ET. Detailed correction procedures are given in the SI.

Note that the instrumental response function (IRF) is close to fluorescence decay curve of ir(btp)_2_acac doped SPF sample, therefore, one may think that intrinsic fluorescence response from the sample will be largely distorted by IRF so that accurate SE lifetime can not be obtained. However, it has been indicated that lifetime down to 1/10 of FWHM (full width at half maximum) of IRF can still be recovered via iterative reconvolution process^55^. The FWHM of IRF is about 80 ps in our detector system, implying that lifetime down to 8 ps can be recovered via iterative reconvolution process. As a result, the fluorescence lifetime of Ir(btp)_2_acac doped SPF_LMW_ (36 ps) obtained in our study is reasonable.

### The determination of η_ET_^T^

Similar to η_ET_^S^, η_ET_^T^ can be expressed by τ_T_^Hd^ and τ_T_^H0^ as η_ET_^T^ = 1 − (τ_T_^Hd^/τ_T_^H0^), where τ_T_^Hd^ and τ_T_^H0^ are TE lifetimes of the polymer host in a presence and absence of phosphor, respectively^51^. Owing to the non-emissive property of TE in the fluorescent polymer, the PIA technique with reflection geometry was employed to determine the TE lifetime of fluorescent polymer^56^, in which a decrease (ΔR) in the intensity of reflection probe beam (R) was recorded (see methods and SI for experimental details). The TE lifetime can be obtained by modeling dependence of fractional change in intensity of reflection probe beam (ΔR/R) on the modulation frequency (ω) with the following equation^57^:

4 $$-{(\frac{{\rm{\Delta }}R}{R})}_{X}={\rm{Re}}(\frac{{C}_{T}^{H0}}{1+{(i\omega {\tau }_{T}^{H0})}^{{\alpha }_{H0}}})$$

**Figure 4:**
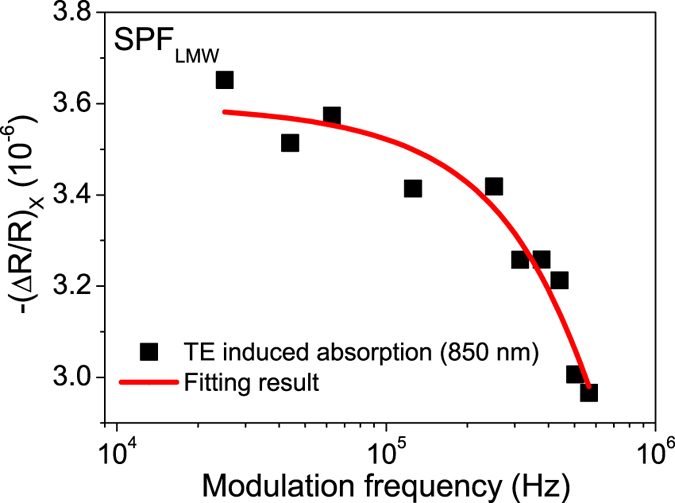
Modulation frequency dependence of PIA signal (850 nm) for SPF_LMW_ at room temperature. The solid line shows fit to Equation 4. The average τ_T_^H0^ and C_T_^H0^ (over three samples) are about 680 ns and 3.67 × 10^−6^ for SPF_LMW_, respectively. The PIA measurement was carried out in device geometry with the structure ITO/PEDOT:PSS/emitting layer/TPBI/Ca/Al.

Here, (ΔR/R)_X_ is the PIA signal recorded in the X channel of dual channel lock-in amplifier, τ_T_^H0^ is “mean lifetime” of TE and α_H0_ is a dispersion parameter that is related to the lifetime distribution function. C_T_^H0^ is a constant proportional to steady state TE concentration in SPF. Figure 4 shows the modulation frequency dependence of TE induced absorption of SPF at room temperature. By fitting frequency dependence of TE induced absorption with Equation 4, we can obtain τ_T_^H0^ of 680 ns accompanying with a C_T_^H0^ of 3.67 × 10^−6^ for pure SPF_LMW_ (these values are an average of three samples). For SPF_LMW_, average τ_T_^Hd^ is about 239 ns from TREL measurement with the aid of Equation 1; therefore, η_ET_^T^ is about 64% in this blend system. The reason for this high η_ET_^T^ is that the hole/exciton blocking layer TPBI can increase the residence time of SPF TE in the luminescent region and thus enhance η_ET_^T^ ^6^. Unlike η_ET_^S^, the correction for η_ET_^T^ is not necessary since TE decay kinetic of the polymer is relatively unaffected in spite of the presence of phosphor dopant^37^.

### χ_S_ in SPF based PLED

**Figure 5:**
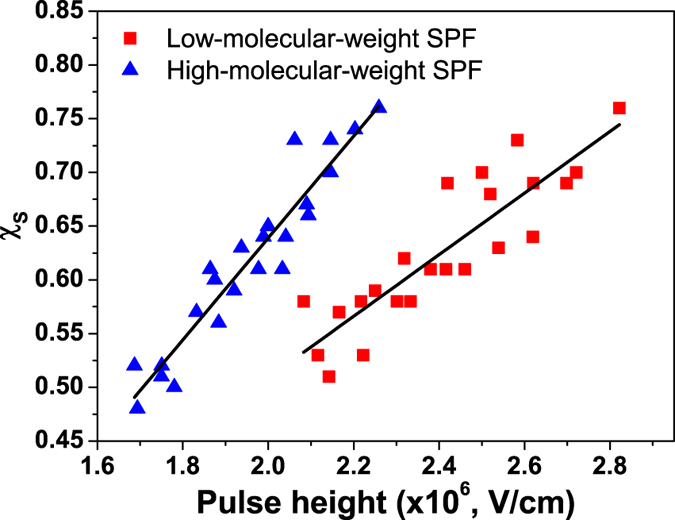
χ_S_ versus pulse height in the SPF based PLEDs. The solid lines are for a guide to eyes.

Given previous measurement of TREL and calculations of η_ET_,_C_^S^ and η_ET_^T^, Fig. 5 shows χ_S_ in the SPF based PLED for each molecular weight. χ_S_ was found to range from 50% to 76%, depending on applied voltage. Higher applied voltage leads to larger χ_S_. This field-dependent χ_S_ can be suggestive of increased probability of SE generation through TTA owing to higher TE concentration at higher applied voltage which promotes the chance of collision between them^18^. It has also been reported that the delayed fluorescence in the polyspirobifluorene is dominated by bimolecular triplet annihilation^58^, supporting that TTA can be a potential way to recycling TE in our SPF.

Although there is no TTA term in our theoretical model (see SI for detailed derivations), the explanation of this field-dependent χ_S_ through TTA is not inappropriate as revealed below. The triplet population on the polymer chains is very high immediately after the electrical excitation in our device. Therefore, the initial quenching of polymer TE can be expected to occur via bimolecular TTA due to high triplet population. Our viewpoint can be supported by previous PIA study on triplet kinetics of isolated polyspirobifluorene polymer chains in dilute solution, in which the triplet is observed to have an initial fast decay component due to bimolecular TTA at high triplet population^59^. Furthermore, from the excitation intensity dependence of the triplet signal, the TTA within a isolated polyspirobifluorene chain in dilute solution can occur in less than 100 ns^59^. Therefore, a more faster TTA rate can be expected in our study due to the contact of individual chains in the sold film allowing more annihilation reactions to take place.

From the above discussion, the time scale of TTA in our system can be significantly less than 100 ns, which is faster than the timescale of triplet-triplet ET and triplet monomolecular decay (~several hundreds ns). Besides, the singlet-singlet ET is instantaneous (~several tens ps) relative to the timescale of triplet-triplet ET (~several hundreds ns) as mentioned above. As a result, the polymer TE that is quenching by TTA can be considered in the initial TE concentration in the polymer, and the formation of extra polymer SE through TTA can be included in the initial TE concentration generated in the phosphor. Therefore, no additional term related to TTA is needed in our theoretical model.

Since TTA is controlled by the diffusion of TE, any intervention in TE diffusion will result in insignificant contribution of TTA to χ_S_. For example, on the basis of separately measured SE and TE formation rates, χ_S_ in poly(2-methoxy-5-(3′,7′-dimethyl)-octyloxy-p-phenylene-vinylene) (OC_1_C_10_-PPV) based PLED was determined to be 83 ± 7%, independent of driving voltage^15^. Their experiment was carried out at 10 K, in which TE could be immobilized such that the probability of TTA is largely reduced, leading to voltage-independent χ_S_. Similarly, no significant variation in χ_S_ with electric field was observed in the PFO based polymer with randomly copolymerized triarylamine (TAA) monomer units (7.5 mol %)^19^. In this copolymer, hole transport takes place via states localized predominantly on the amines^60^. As a result, we speculate that more efficient triplet-charge annihilation (it can lead to decrease in density of TE)^28^ will occur between TE on the PFO and hole on the TAA, leading to suppression of TTA effect.

Surprisingly, the molecular weight of conjugated polymer can also affect χ_S_. The χ_S_ in SPF_HMW_ increases more rapidly with electric field as compared to that in SPF_LMW_. This result also suggests that TTA takes place within PLED since a longer-chain SPF_HMW_ can accommodate more TEs as compared to that in a shorter-chain SPF_LMW_, thereby increasing the probability of intramolecular TTA within the polymer chain. Our viewpoint can be supported by previous studies of molecular weight dependent delayed fluorescence of non-conjugated polymer, in which intensity of delayed fluorescence arising from TTA increases with molecular weight of polymer^61, 62^. The molecular weight dependent χ_S_ here implies that we can manipulate χ_S_ possibly via chain orientation of conjugated polymers, which doesn’t exist in the OLED and is in need of further investigation. Through this fundamental study, it suggests that conjugated polymers are possibly competitive in practice to small molecules as highly efficient luminescent materials.

One question may be raised, the molecular weight of SPF_HMW_ is higher than that of SPF_LMW_ only by a factor of about 1.15, there is hardly significant difference in molecular weight so that tunable χ_S_ should be tenuous. However, as reported by Pasch *et al*.^62^, the intensity of delayed fluorescence arising from TTA can be enhanced by a factor of 1.63 even if the molecular weight of non-conjugated polymer (poly(2-naphthyl methacrylate)) is slightly increased by a factor of 1.45. As a result, the molecular-weight-dependent χ_S_ in our study can be expected. Besides, as compared with previously reported non-conjugated polymers (poly(N-vinylcarbazole) and poly(2-naphthyl methacrylate))^61, 62^, SPF will be expected to have more fast TE migration and thus more efficient TTA due to its conjugated structure. Furthermore, the PDI of SPF_LMW_ is larger than that of SPF_HMW_ by a factor of 1.23 (1.66 vs. 1.34); this result reflects that more shorter-chain SPF in the SPF_LMW_ might also lead to a decreased probability of TTA. Therefore, SPF_LMW_ gives smaller χ_S_.

It have been reported that a very small energy gap (~14 meV) occurs in charge transfer based singlet and triplet states (^1^CT and ^3^CT) of polyspirobifluorene due to orthogonal nature between highest occupied molecular orbital (HOMO) and lowest unoccupied molecular orbital (LUMO)^63, 64^. As a result, the ^3^CT generated from decay of upper triplet state (created by TTA) can convert to ^1^CT via RISC, leading to luminescence through radiative decay of ^1^CT (This process is called TADF)^65^. Therefore, there are indeed two possible ways for extra SE generation by TTA in our SPF. First, the up-converted triplet state (created by TTA) is close to that of the singlet one, resulting in p-type delayed fluorescence^66^. The second is via TADF, in which the up-converted triplet state formed via TTA can decay to ^3^CT by electron transfer, and ^3^CT then converts to ^1^CT via RISC due to very small energy gap. The generated ^1^CT then produces photons through its radiative decay. Therefore, the probability of generation of SE from TTA could be increased. This is also an additional source for SE fraction.

If we assume the condition for no TTA involved is met for the lower limit of our χ_S_ (these values are obtained near the turn on voltage of pulse-drived device), χ_S_ through charge recombination is about 50%. The χ_S_ greater than statistical limit 25% in charge recombination process can support the previously proposed scenario for exciton formation^14^: the formation rate of TE from triplet polaron pairs (PP) could be slower than interconversion rate from triplet PP to singlet PP. Therefore, triplet PP has the probability to convert into singlet PP. Along with faster formation rate from singlet PP to SE relative to that from triplet PP to TE, the χ_S_ can thus exceed statistical limit 25%.

The interconversion rate between triplet PP and singlet PP depends on energy gap between these two states, and an appreciable energy gap will lead to inefficient conversion rate^67^. In the absence of this process, χ_S_ through charge recombination will be forced to 25% as the upper limit, regardless of the difference in formation rates of SE and TE^67^. Since this gap increases with decreasing interchain distance^68^, we can expect a larger energy gap (and thus inefficient interconversion) in the aggregates of MEH-PPV due to its intuitively close interchain distance relative to its amorphous parts, leading to the reported charge-recombination χ_S_ of MEH-PPV approaching statistical limit 25%^10^. This result also suggests that, in addition to formation of emitting species with low luminescence efficiency due to close interchain distance of polymer (such as aggregates in MEH-PPV), the close interchain distance could also have detrimental effect on the initial SE formation as discussed above.

### Conclusion

In summary, via the measurement of phosphor transient emission of electrophosphorescent PLED and separately obtained ET efficiencies, we found that χ_S_ in the SPF based PLED ranging between 50% and 76%. The 50% is contributed by charge recombination process and the increment in χ_S_ with applied voltage was observed. The molecular weight dependence of χ_S_ process also suggests readily-tailored χ_S_ property for conjugated polymer, which does not exist in the small molecule. Besides, χ_S_ greater than the statistical limit 25% in charge recombination process is in agreement with the efficient interconversion between triplet and singlet PPs and with larger formation rate of SE relative to that of TE. To sum up, more efforts are needed to investigate structure-property relationships for this most important parameter in electrofluorescence from conjugated polymers.

## Methods

### Materials

The polymer spiropolyfluorene (SPF) was synthesized according to previous report^69^. The bis(2-benzo[b]thiophen-2-yl-pyridine)(acetylacetonate)iridium(III) (Ir(btp)_2_acac) was purchased from Luminescence Technology (Taiwan) and used without further purification.

### Instrumentation

Ultraviolet-visible (UV-Vis) and photoluminescence (PL) spectra were measured using an UV-Vis-Near IR spectrometer (Perkin-Elmer, Lambda 19) and a fluorescence spectrometer (Jobin Yvon Horiba, Fluoromax-3), respectively. Gel permeation chromatography (from Waters) assembled with a UV detector and three columns in series (Styragel HR2~4 from Waters) was used to measure molecular weight distributions relative to polystyrene standards at 40 °C. The calibration curve was determined by use of seven specified standards with molecular weights from 1240 to 5.32 × 10^5^. Tetrahydrofuran (THF) was used as carrier solvent at flow rate 1.0 mL/min.

### Device fabrication

The fabrication procedures for the device are as follows: indium-tin oxide (ITO) glass was exposed to oxygen plasma at a power of 50 W and a pressure of 200 mTorr for 5 min. A thin layer (20 nm) of poly(3,4-ethylenedioxythiophene):poly(styrenesulfonate) (PEDOT:PSS) (Clevios PVP AI 4083 from Heraeus, the resistivity is 500–5000 Ω cm) was spin-coated on the treated ITO as a hole injection layer. On top of the PEDOT:PSS layer, an emitting layer was spin-coated from its solution in THF. The 1,3,5-tris(2-henylbenzimidazolyl)benzene (TPBI) layer (25 nm), which was used as a hole/exciton blocking layer, was grown by thermal evaporation in a vacuum of 2 × 10^−6^ Torr. Finally, a thin layer of calcium (about 4 nm) covered with a layer of aluminum for a bipolar device was deposited in a vacuum thermal evaporator through a shadow mask at a vacuum of 2 × 10^−6^ Torr.

### Time-resolved electroluminescence (TREL)

For TREL measurement, voltage pulses with duration of 200 ns and repetition rate of 100 Hz from a pulse generator (Avtech AV1015-B) was applied to the device. The emitted light through a bandpass filter was measured by a photomultiplier tube (Hamamatsu) connected to a 350 MHz oscilloscope (LeCroy). All samples were measured in a vacuum environment under room temperature.

### Time-resolved photoluminescence (TRPL)

The device structure rather than thin film spin-coated on the quartz was employed in TRPL measurement. The TRPL measurement was carried out in a vacuum environment under room temperature. The photoluminescence decay curves of SPF with and without phosphor were measured by a time-correlated single photon counting (TCSPC) system with a microchannel plate photomultiplier tube (Hamamatsu Photonics R3809U-50) and a spectrometer (Edinburgh Lifespec-ps with TCC900 data acquisition card). Excitation pulse for the TCSPC experiment was provided by a frequency doubled output (harmonic generator, Inrad 5-050) of a mode-locked Ti-Sapphire laser (Coherent Mira 900) pumped by a solid-state diode-pumped laser (Coherent Verdi V-10). The repetition rate was reduced to 3.8 MHz by a pulse picker (Coherent Model 9200) between Mira 900 and In-rad 5-050.

### Photo-induced absorption (PIA)

In the present work, PIA measurement was performed in the device structure. The samples were subjected to measurement under a vacuum at room temperature. A 405 nm continuous wave (CW) laser (pump beam) near the UV-Vis maximum of SPF (388 nm) was modulated by an electro-optic modulator and used to excite the sample and generate singlet exciton (SE). The triplet exciton (TE) was then formed by intersystem crossing (ISC) from SE, which was probed by an 850 nm CW laser close to the maximum of TE induced absorption (812 nm)^37^. The probe beam was incident from transparent ITO side through polymer layer to metal side and reflected by the metal cathode of device. A decrease in the intensity of the reflection probe beam (ΔR) was measured by a combination of silicon photodetector and dual channel lock-in amplifier (Stanford Research Systems, SR830), in which a monochromator was placed in front of photodetector to reduce PL signal due to pump beam excitation and any remaining PL signal was measured by blocking the probe beam and subtracted from the total signal. The ΔR was then normalized by the intensity of reflection probe beam (R) (it was measured using the multimeter) to give (ΔR/R). Induced absorption due to TE showed a negative signal in the X channel ((ΔR/R)_X_ < 0) and a positive signal in the Y channel ((ΔR/R)_Y_ > 0) if the phase of dual channel lock-in amplifier was set such that the PL signal of SPF exhibited entirely a positive value in the X channel^70^.

## Electronic supplementary material

Supplementary Information
