# Supplementary material for: Singlet Exciton Fraction in Electroluminescence from Conjugated Polymer
Source: Sci Rep. 2017 Jun 6;7:2889. doi: 10.1038/s41598-017-02115-2 (PMC5460123; doi:10.1038/s41598-017-02115-2)
Supplement: Supplementary file 1 — Supplementary Information [file 41598_2017_2115_MOESM1_ESM.pdf]

Supplementary Information

Singlet Exciton Fraction in Electroluminescence from Conjugated Polymer

Tzu-Hao Jen[1] & Show-An Chen[1]

[1] Department of Chemical Engineering and Frontier Research Center on Fundamental and Applied Sciences of Matters, National Tsing-Hua University, Hsinchu 30013 Taiwan (ROC). Correspondence and requests for materials should be addressed to S.-A. C. (email: sachen@che.nthu.edu.tw)

Supplementary Figures

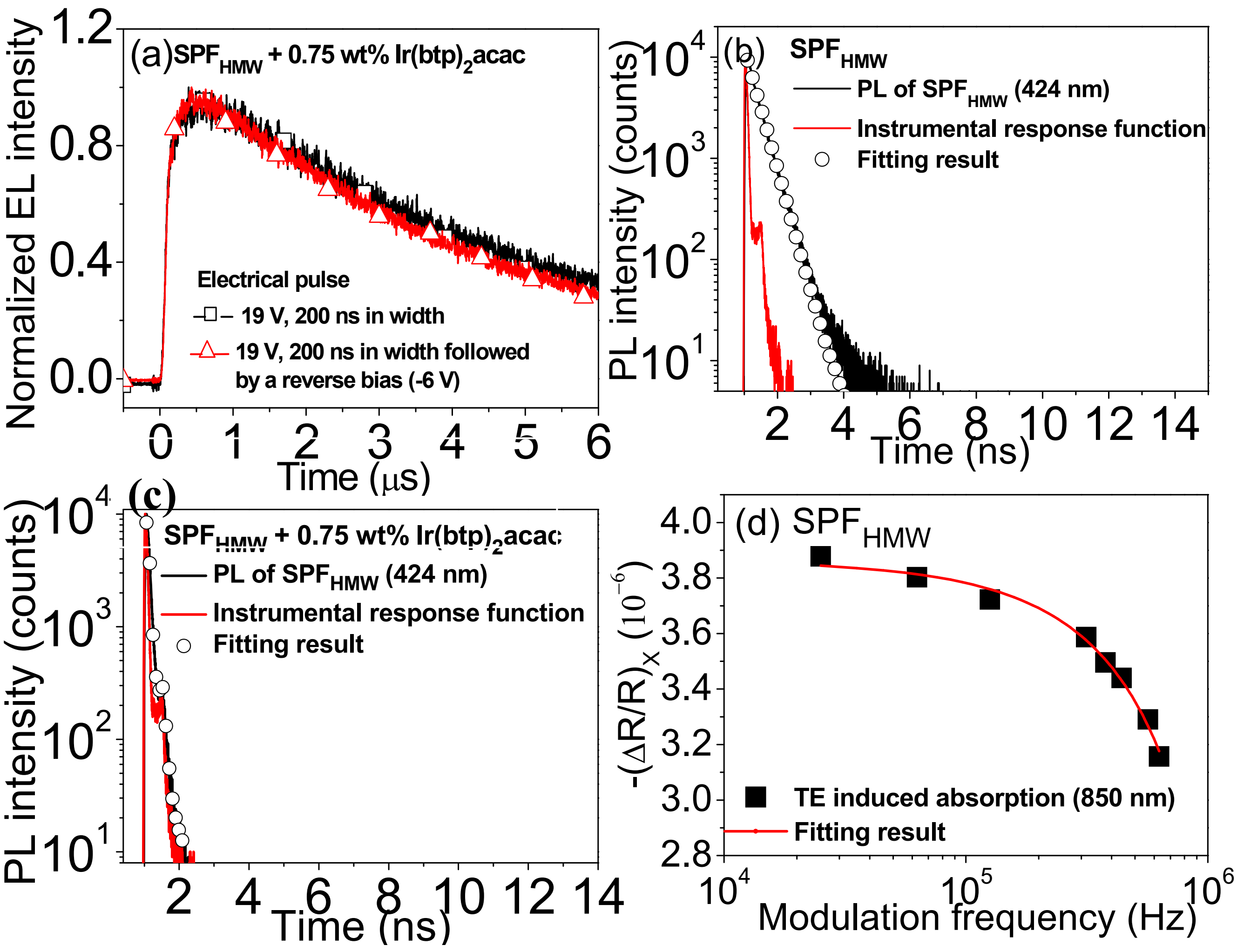

Supplementary Figure 1. (**a**) TREL and (**c**)TRPL of Ir(btp)$_2$acac doped SPF$_{HMW}$. (**b**)TRPL and (**d**) PIA of SPF$_{HMW}$. These measurements were carried out in device geometry with the structure: ITO/PEDOT:PSS/emitting layer/TPBI/Ca/Al.

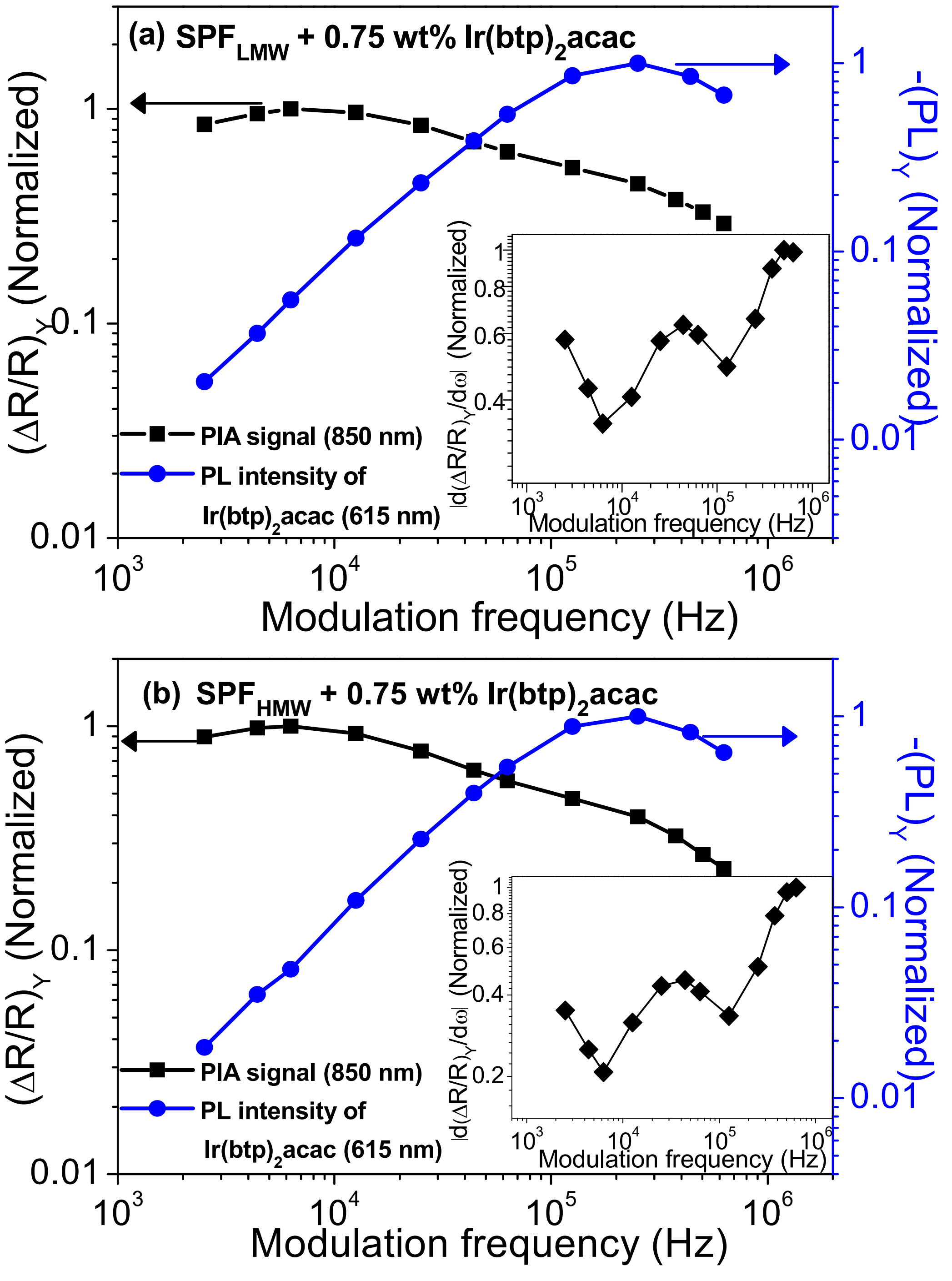

Supplementary Figure 2. Modulation frequency dependence of PIA signal (850 nm) of $Ir(btp)_2acac$ doped (**a**) SPF_{LMW} and (**b**) SPF_{HMW} at 78 K. The dependences of phosphorescence intensity (615 nm) of $Ir(btp)_2acac$ ($-(PL)_Y$) upon the modulation frequency for these two sample are also shown for comparison. The dependence of $|d(\Delta R/R)_Y/d\omega|$ upon modulation frequency is provided in the inset.

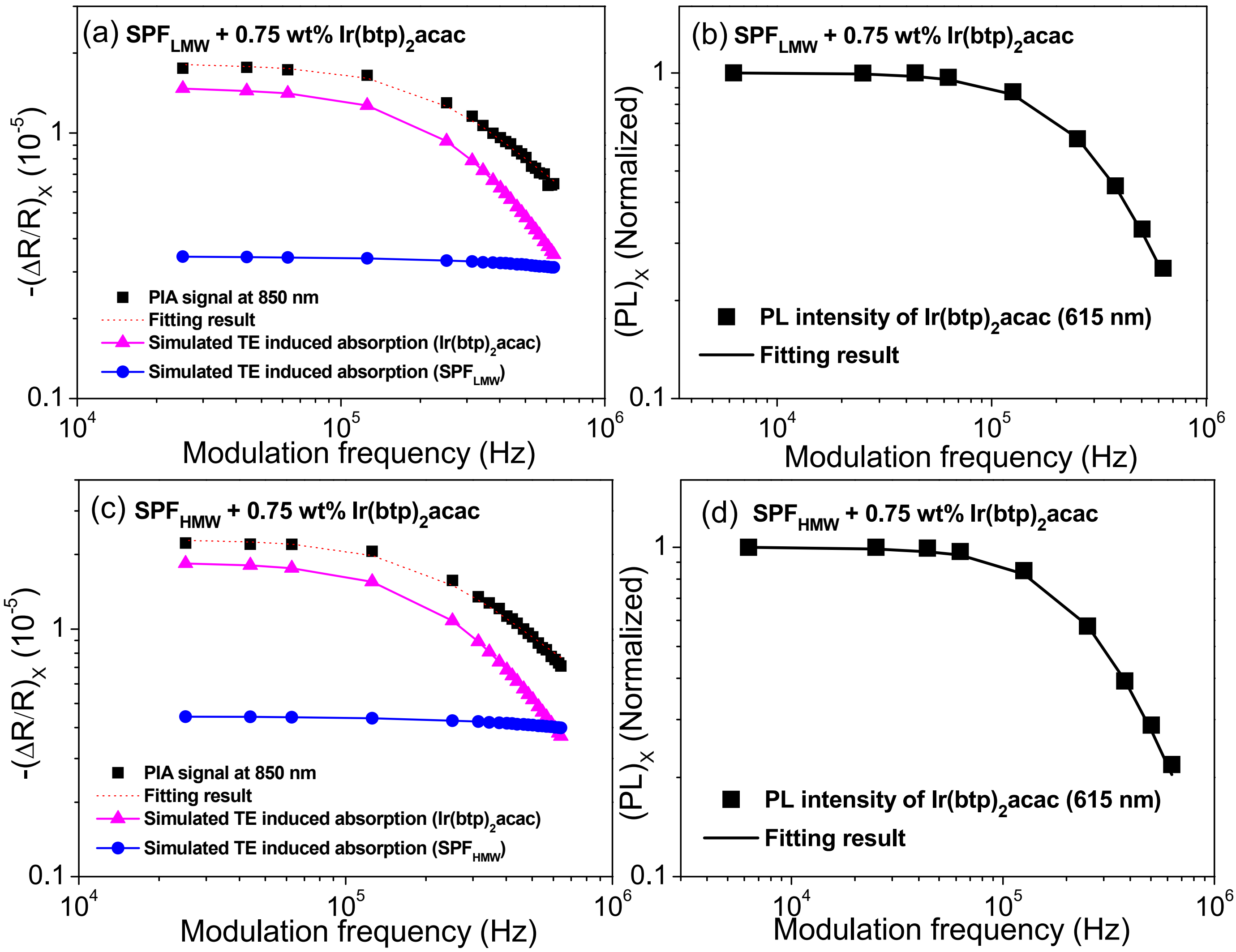

Supplementary Figure 3. (**a**) The modulation frequency dependence of PIA signal (850 nm) of Ir(btp)$_2$acac doped SPF$_{LMW}$ at room temperature. The red line represents a fit of Equation S8 to the data. The simulated TE induced absorption of Ir(btp)$_2$acac and SPF$_{LMW}$ are also shown for comparison. (**b**) The dependence of PL intensity (615 nm) of Ir(btp)$_2$acac (PL)x as a function of modulation frequency at the same sample at room temperature. The solid line is a fit of equation $(PL)x=Re(K/(1+(i\omega\tau_T{}^{G})^{\alpha_G}))$. Those for SPF$_{HMW}$/Ir(btp)$_2$acac blend system are provided in (**c**) and (**d**).

Supplementary Tables

Supplementary Table 1. χ_S in the SPF_{LMW} based PLED.

Pulse height (V/cm)	A/C	([S]/[T])[a]	χ_S[b]
2.083×10^6	1.67	1.41	0.58
2.116×10^6	1.36	1.15	0.53
2.142×10^6	1.27	1.07	0.51
2.166×10^6	1.59	1.34	0.57
2.217×10^6	1.68	1.41	0.58
2.222×10^6	1.38	1.16	0.53
2.250×10^6	1.75	1.47	0.59
2.301×10^6	1.69	1.42	0.58
2.318×10^6	1.95	1.64	0.62
2.333×10^6	1.65	1.39	0.58
2.380×10^6	1.92	1.62	0.61
2.416×10^6	1.92	1.62	0.61
2.419×10^6	2.65	2.23	0.69
2.460×10^6	1.9	1.6	0.61
2.500×10^6	2.85	2.4	0.70
2.520×10^6	2.53	2.13	0.68
2.539×10^6	2.09	1.76	0.63
2.583×10^6	3.25	2.74	0.73
2.619×10^6	2.15	1.81	0.64
2.620×10^6	2.7	2.27	0.69
2.698×10^6	2.65	2.23	0.69
2.721×10^6	2.88	2.43	0.70
2.822×10^6	3.79	3.19	0.76

[a]The ET efficiencies of SE ($\eta_{ET,C}^{S}$) and TE (η_{ET}^{T}) in SPF_{LMW}/Ir(btp)$_2$acac system are 76% and 64%, respectively, and [S]/[T] can be expressed in terms of A/C, $\eta_{ET,C}^{S}$ and η_{ET}^{T} as the expression [S]/[T] = (A/C)×(η_{ET}^{T}/$\eta_{ET,C}^{S}$). [b]χ_S can be expressed in terms of [S]/[T] as the expression χ_S = ([S]/[T])/(1+([S]/[T])).

Supplementary Table 2. χ_S in the SPF_{HMW} based PLED.

Pulse height (V/cm)	A/C	([S]/[T])[a]	χ_S[b]
1.687×10^6	1.58	1.11	0.52
1.694×10^6	1.34	0.94	0.48
1.750×10^6	1.53	1.08	0.51
1.751×10^6	1.57	1.11	0.52
1.780×10^6	1.42	1	0.50
1.832×10^6	1.96	1.38	0.57
1.864×10^6	2.28	1.61	0.61
1.875×10^6	2.16	1.52	0.60
1.884×10^6	1.85	1.3	0.56
1.920×10^6	2.05	1.45	0.59
1.937×10^6	1.91	1.35	0.57
1.937×10^6	2.42	1.71	0.63
1.977×10^6	2.23	1.57	0.61
1.989×10^6	2.53	1.78	0.64
2.000×10^6	2.68	1.89	0.65
2.033×10^6	2.26	1.59	0.61
2.041×10^6	2.58	1.82	0.64
2.062×10^6	3.98	2.81	0.73
2.090×10^6	3	2.12	0.67
2.094×10^6	2.86	2.02	0.66
2.146×10^6	3.86	2.72	0.73
2.146×10^6	3.36	2.37	0.70
2.203×10^6	4.1	2.89	0.74
2.259×10^6	4.67	3.29	0.76

[a]The ET efficiencies of SE ($\eta_{ET,C}{}^{S}$) and TE ($\eta_{ET}{}^{T}$) in SPF_{HMW}/Ir(btp)$_2$acac system are 78% and 55%, respectively, and ([S]/[T]) can be expressed in terms of A/C, $\eta_{ET,C}{}^{S}$ and $\eta_{ET}{}^{T}$ as the expression ([S]/[T]) = (A/C)×($\eta_{ET}{}^{T}$/$\eta_{ET,C}{}^{S}$).
[b]χ_S can be expressed in terms of [S]/[T] as the expression χ_S = ([S]/[T])/(1+([S]/[T])).

Supplementary Notes

Supplementary Note 1. The theoretical equation for singlet exciton fraction (χ_S) measurement. In the phosphor doped polymer light-emitting diode (PLED), the triplet state of phosphor guest can be effectively generated by two excitation mechanisms: (a) singlet exciton (SE) and triplet exciton (TE) formed in the host under electrical excitation transfer to phosphor via singlet-singlet and triplet-triplet energy transfer (ET), respectively; and SE in phosphor is converted to TE by very efficient (~100%) and fast (~fs) intersystem crossing (ISC)[1]. (b) Holes and electrons injected from the anode and cathode directly recombine on the phosphor through the charge trapping. Therefore, the rate equation for concentration of TE in the phosphor $[T]_G$ can be described by the following equation:

$$\frac{d[T]_G}{dt} = k_{ET}^T [T]_H - k_{R,G}^T [T]_G - k_{NR,G}^T [T]_G \qquad \text{(S1)}$$

Where $[T]_H$ is concentration of TE in the host and k_{ET}^T is rate constant of triplet-triplet ET from host to guest. $k_{R,G}^T$ and $k_{NR,G}^T$ are radiative and non-radiative decay rate constants of guest TE, respectively. The second and third terms on the right side of Equation S1 describe TEs in the phosphor are consumed due to radiative and non-radiative decay processes, and those populated through the triplet-triplet ET process is described by the first term. As to be shown later, the contribution of SE transfer from host to phosphor will be included in the initial TE concentration generated in the phosphor because singlet-singlet ET is instantaneous (~several ten ps) relative to the timescale of triplet-triplet ET (~several hundred ns) as mentioned in the main text. Therefore, no additional term related to phosphor TE generated through singlet-singlet ET from host is needed in Equation S1. Similarly, the contribution of charge trapping to phosphor TE can also be included in the initial TE concentration generated in the phosphor due to very fast timescale of electron-hole recombination (~ps)[2] as compared to that of triplet-triplet ET (~several hundred ns). To solve Equation S1, it is intuitively required to know time dependent TE in the host $[T]_H$. The rate equation for $[T]_H$ can be expressed as follows:

$$\frac{d[T]_H}{dt} = -k_{ET}^T [T]_H - k_{R,H}^T [T]_H - k_{NR,H}^T [T]_H \qquad \text{(S2)}$$

Where k_{ET}^T is rate constant of triplet-triplet ET from host to guest. $k_{R,H}^T$ and $k_{NR,H}^T$ are radiative and non-radiative decay rate constants of host TE. The three terms on the right side of the Equation S2 are corresponding to consumption of TE in the host by triplet-triplet ET from host to guest (first term), radiative and non-radiative decay processes of host TE (second and third terms). With the initial condition $[T]_H(0) = [T]_{H,ini}$ (initial TE concentration in the host), Equation S2 is readily solved and yields the following functional form:

$$[T]_H = [T]_{H,ini} \cdot \exp[-(k^T_{R,H} + k^T_{NR,H} + k^T_{ET}) \cdot t] \quad \text{(S3)}$$

Plug Equation S3 into Equation S1 and assume $[T]_G(0) = [T]_{G,ini}$ (initial TE concentration generated in the phosphor), we can obtain time dependent $[T]_G$ as follows:

$$[T]_G = A \cdot \exp\left(\frac{-t}{\tau^G_T}\right) + B \cdot \left[\exp\left(\frac{-t}{\tau^G_T}\right) - \exp\left(\frac{-t}{\tau^{Hd}_T}\right)\right] \quad \text{(S4)}$$

and

$$A = [T]_{G,ini} \qquad B = k^T_{ET} \cdot [T]_{H,ini} \cdot \left(\frac{\tau^{Hd}_T \cdot \tau^G_T}{\tau^G_T - \tau^{Hd}_T}\right)$$

Here, $\tau_T^{Hd}=(k_{R,H}^T+k_{NR,H}^T+k_{ET}^T)^{-1}$ and $\tau_T^G=(k_{R,G}^T+k_{NR,G}^T)^{-1}$, which are TE lifetimes of conjugated polymer and phosphor, respectively. Besides, we further define C as below:

$$C = B \cdot \left(1 - \frac{\tau^{Hd}_T}{\tau^G_T}\right) = \left(\frac{k^T_{ET}}{k^T_{R,H} + k^T_{NR,H} + k^T_{ET}}\right) \cdot [T]_{H,ini} = \eta^T_{ET} \cdot [T]_{H,ini} \quad \text{(S5)}$$

Where η_{ET}^T is triplet-triplet ET efficiency. If we divide A by C, and note that $[T]_{G,ini}$ comes from direct charge trapping on the phosphor and SE transfer from conjugated polymer by singlet-singlet ET, we can obtain:

$$\frac{A}{C} = \frac{[T]_{G,ini}}{\eta^T_{ET} \cdot [T]_{H,ini}} = \frac{\eta^S_{ET} \cdot [S]_{H,ini} + [T]_{G,CT}}{\eta^T_{ET} \cdot [T]_{H,ini}} \quad \text{(S6)}$$

Here, η_{ET}^S is efficiency of singlet-singlet ET and $[T]_{G,CT}$ represents the phosphor TE concentration coming from charge trapping. $[S]_{H,ini}$ is the initial SE concentration generated in the conjugated polymer. If appropriate phosphor and conjugated polymer are selected so that charge trapping is negligible, then from Equation S6 we can obtain:

$$\frac{A}{C} = \frac{[T]_{G,ini}}{\eta^T_{ET} \cdot [T]_{H,ini}} = \frac{\eta^S_{ET} \cdot [S]_{H,ini}}{\eta^T_{ET} \cdot [T]_{H,ini}} \quad \text{(S7)}$$

$[S]_{H,ini}/[T]_{H,ini}$ is the ratio of SE to TE in the conjugated polymer, which is corresponding to [S]/[T] in the main text.

Supplementary Note 2. Transient electroluminescence (TREL) 、 transient photoluminescence (TRPL) and photoinduced absorption (PIA) of high-molecular-weight SPF (SPF_{HMW}) related systems. Supplementary Fig. 1a shows TREL of $Ir(btp)_2acac$ at 610 nm in $Ir(btp)_2acac$ doped SPF_{HMW}. A rise (~several hundred ns) of transient phosphorescence and negligible effect of reverse bias applied on transient EL emission of $Ir(btp)_2acac$ indicate that triplet-triplet ET takes place in the present system[3]. Supplementary Fig. 1b shows TRPL of SPF_{HMW} at 424 nm when it was excited with a pulse of laser light (387 nm); and that in the presence of $Ir(btp)_2acac$ is given in the inset. For SPF_{HMW}, the average SE lifetime is reduced to 48 ps (τ_S^{Hd}) from 358 ps (τ_S^{H0}) when the polymer is doped with $Ir(btp)_2acac$, resulting in singlet-singlet ET efficiency (η_{ET}^S) of 87% based on relation $\eta_{ET}^S = 1-(\tau_S^{Hd}/\tau_S^{H0})^4$. After correction for external heavy atom effect induced by phosphor as to be shown later, the corrected singlet-singlet ET efficiency ($\eta_{ET,C}^S$) is about 78%. Modulation frequency dependence of PIA signal (850 nm) of SPF_{HMW} at room temperature is also provided in Supplementary Fig. 1c The τ_T^{H0} (TE lifetime of polymer) and C_T^{H0} (a constant proportional to steady state TE concentration in polymer) can be obtained by fitting the experimental data to Equation 4 in the main text and are about 574 ns and 4.3×10^{-6} (these values are averages of three samples) for SPF_{HMW}, respectively. Along with an average τ_T^{Hd} (TE lifetime of polymer upon phosphor doping) of 257 ns determined from TREL measurement, η_{ET}^T is about 55% in this blend system based on similar relation $\eta_{ET}^T = 1-(\tau_T^{Hd}/\tau_T^{H0})^4$.

Supplementary Note 3. Correction for efficiency of singlet-singlet ET. It has been reported that inclusion of phosphorescent heavy-metal complex can enhance ISC rate of host polymer[5], leading to overestimation of η_{ET}^S. Therefore, we have to evaluate this heavy atom effect to obtain $\eta_{ET,C}^S$ in the present system, which can be aided by observing the change in number of TE in the polymer host in a presence and absence of the phosphor guest. In the following, we will show how correction procedures are performed.

The wavelength of probe beam employed to detect TE concentration of SPF is 850 nm, which is near the maximum of TE induced absorption for SPF (812 nm)[5]. The wavelength of pump beam is 405 nm, it is also near the maximum of UV-Vis (388 nm) for SPF. When the $Ir(btp)_2acac$ is doped into SPF, we have to check whether $Ir(btp)_2acac$ also contributes to PIA signal at the wavelength of TE absorption of SPF. If it does contribute to PIA signal, we have to add an additional term in Equation 4 in the main text to describe PIA due to $Ir(btp)_2acac$ inclusion. Supplementary Fig. 2a displays the modulation frequency dependence of PIA signal recorded in the Y channel of dual channel lock-in amplifier $(\Delta R/R)_Y$ for $Ir(btp)_2acac$ doped SPF_{LMW} at 78 K. Here, $(\Delta R/R)_Y$ was observed for the following reason: for an excited state species with lifetime τ_{ex}, a maximum $(\Delta R/R)_Y$ can occur in $\omega\sim1/\tau_{ex}$[6], therefore, if there are two excited state species with different lifetimes existed in the sample, two peaks should

be observed in $(\Delta R/R)_Y$. As can be seen in the Supplementary Fig 2a, a peak and a shoulder around 6-7 kHz and 100-200 kHz appeared in the $(\Delta R/R)_Y$ (or more clearly from the ｜d(ΔR/R)Y/dω｜ versus ω in the inset, in which we can see two local minima in the corresponding positions), indicating existence of two excited state species within SPF_{LMW}/Ir(btp)$_2$acac system. We assign them as arising from TE induced absorption of SPF_{LMW} and Ir(btp)$_2$acac, respectively. The judgment for shoulder at around 100-200 kHz resulting from induced absorption of Ir(btp)$_2$acac TE can be supported by observing dependence of phosphorescence intensity of Ir(btp)$_2$acac (615 nm) upon the modulation frequency at the same sample (Supplementary Fig. 2a). The setup and geometry of this phosphorescence measurement is the same as PIA except no probe beam is involved. The modulation pump beam illuminates the sample and gives rise to modulation change in phosphorescence intensity of Ir(btp)$_2$acac, which can be detected by a combination of silicon photodetector and dual channel lock-in amplifier. $(PL)_Y$ indicates the phosphorescence intensity of Ir(btp)$_2$acac recording in the Y channel of dual channel lock-in amplifier when the phase of dual channel lock-in amplifier is set such that the PL signal of SPF exhibits entirely a positive value in the X channel. Similar to $(\Delta R/R)_Y$, $-(PL)_Y$ should have a maximum at the position $\omega_{max}\sim 1/\tau_T^G$, in which τ_T^G is the lifetime of Ir(btp)$_2$acac TE. As can be seen in the Supplementary Fig. 2a, the maximum of $-(PL)_Y$ appears at about 200 kHz, which is close to the shoulder position of $(\Delta R/R)_Y$. In other words, the excited state specie that contributes to induced absorption around 100-200 kHz in $(\Delta R/R)_Y$ should have lifetime similar to Ir(btp)$_2$acac TE, implying that this induced absorption can be ascribed to Ir(btp)$_2$acac TE. Similar phenomenon was also observed in the Ir(btp)$_2$acac doped SPF_{HMW} (Supplementary Fig. 2b). Therefore, both TEs of Ir(btp)$_2$acac and SPF contribute to the PIA signal at 850 nm.

From the above discussion, we know that the origin of PIA signal at 850 nm in Ir(btp)$_2$acac doped SPF is the summation of TE induced absorption from Ir(btp)$_2$acac and SPF. Therefore, it is intuitively required to add an additional term in Equation 4 in the main text to take account of the contribution of induced absorption from phosphor dopant, which is given by:

$$-\left(\frac{\Delta R}{R}\right)_X = \mathrm{Re}\left(\frac{I_T^G}{1+\left(i\omega\tau_T^G\right)^{\alpha_G}}\right)+\mathrm{Re}\left(\frac{C_T^{Hd}}{1+\left(i\omega\tau_T^{Hd}\right)^{\alpha_{Hd}}}\right) \qquad \text{(S8)}$$

Here, C_T^{Hd} and τ_T^{Hd} are TE concentration (at steady state) and lifetime of polymer upon phosphor doping in comparison to those without phosphor (C_T^{H0} and τ_T^{H0}) in Equation 4. I_T^G and τ_T^G are steady state TE concentration and TE lifetime of phosphor. The first term and second term on the right side of Equation S8 describe TE induced absorption from Ir(btp)$_2$acac and SPF, respectively. Although there are six parameters in the equation above,

some parameters can be determined in advance. By measuring phosphorescence intensity of $Ir(btp)_2acac$ versus modulation frequency at the same sample, one can infer the constants τ_T^G and α_G by fitting data to equation $(PL)x = Re(K/(1+(i\omega\tau_T^G)^{\alpha_G}))$, where K is a proportional constant and $(PL)_X$ is the phosphorescence intensity of $Ir(btp)_2acac$ recorded in the X channel of dual channel lock-in amplifier when phase of dual channel lock-in amplifier is set such that the PL signal of SPF exhibits entirely a positive value in the X channel. This equation is similar to Equation 4 in the main text, except $-(\Delta R/R)_X$ is replaced by $(PL)_X$. Here, $(PL)_X$ was employed to determine τ_{TG} and α_G since PIA signal recorded in the X channel of dual channel lock-in amplifier $-(\Delta R/R)_X$ was used to evaluate the number of TE in the polymer host upon phosphor doping. As shown in Supplementary Fig. 3b,d, τ_T^G and α_G are approximately 3 μs and 0.96 for $Ir(btp)_2acac$ in $SPF_{LMW}/Ir(btp)_2acac$ blend system, and those of $Ir(btp)_2acac$ in $SPF_{HMW}/Ir(btp)_2acac$ are 3.4 μs and 0.96. The τ_T^{Hd} can also be independently obtained by TREL measurement, giving 239 ns and 257 ns for $SPF_{LMW}/Ir(btp)_2acac$ and $SPF_{HMW}/Ir(btp)_2acac$, respectively. Therefore, only three parameters (I_T^G, C_T^{Hd} and α_{Hd}) are adjustable. The red dot lines in Supplementary Fig. 3a,c represent fits of Equation S8 to the data. Besides, simulated TE induced absorption of $Ir(btp)_2acac$ and SPF are also shown for comparison. From these simulations, it shows PIA signal of $SPF/Ir(btp)_2acac$ at room temperature mainly comes from TE induced absorption of $Ir(btp)_2acac$, in which I_T^G (steady state TE concentration of $Ir(btp)_2acac$) is larger than C_T^{Hd} (steady state TE concentration of SPF) by a factor of 4-5 (these values can be evaluated from corresponding TE induced absorption of $Ir(btp)_2acac$ and SPF when ω is close to zero). A mean C_T^{Hd} of 4.1×10^{-6} for $SPF_{LMW}/Ir(btp)_2acac$ was determined from three samples. Along with average C_T^{H0} of 3.67×10^{-6} from the PIA measurement of pure SPF_{LMW}, C_T^{Hd}/C_T^{H0} is equal to 1.12, indicating that TE concentration is increased by a factor of 1.12 due to external heavy atom effect. Similarly, for $SPF_{HMW}/Ir(btp)_2acac$, average C_T^{H0} and C_T^{Hd} are 4.3×10^{-6} and 4.5×10^{-6}, resulting in C_T^{Hd}/C_T^{H0} of 1.05.

Because C_T^{Hd} and C_T^{H0} are proportional to steady state TE concentrations of SPF in a presence and absence of phosphor, respectively, therefore, C_T^{Hd}/C_T^{H0} can be expressed in terms of Φ_{ISC}^{H0}, Φ_{ISC}^{Hd} and η_{ET}^T as shown below by considering TE generation via ISC under photoexcitation and occurrence of triplet-triplet ET upon phosphor doping:

$$\frac{C_T^{Hd}}{C_T^{H0}} = \frac{\Phi_{ISC}^{Hd}\left(1-\eta_{ET}^T\right)}{\Phi_{ISC}^{H0}} \qquad \text{(S9)}$$

Where Φ_{ISC}^{Hd} and Φ_{ISC}^{H0} are ISC efficiencies of phosphor doped and undoped polymer, respectively, and η_{ET}^T is triplet-triplet ET efficiency. In the derivation of Equation S9, we have assumed that there is no difference in initial number of photogenerated SE in polymer

host and that TE absorption cross sections for pure and doped SPF are similar. For $SPF_{LMW}/Ir(btp)_2acac$ blend system, its η_{ET}^{T} and C_T^{Hd}/C_T^{H0} are 64% and 1.12, respectively. Therefore, $\Phi_{ISC}^{Hd}/\Phi_{ISC}^{H0} = 3.1$ from Equation S9, indicating that ISC efficiency is enhanced by a factor of 3.1 due to phosphor doping for present system. The average SE lifetimes for doped (τ_S^{Hd}) and pure (τ_S^{H0}) SPF_{LMW} are 36 ps and 293 ps, respectively. Furthermore, the ISC efficiency of pure SPF is about 4%[5]. Therefore, ISC rates of 3.45×10^{9} s^{-1} (k_{ISC}^{Hd}) and 1.37×10^{8} s^{-1} (k_{ISC}^{H0}) for doped and pure SPF_{LWM} can be determined based on the relation $k_{ISC}^{H0} = \Phi_{ISC}^{H0}/\tau_S^{H0}$ (or $k_{ISC}^{Hd} = \Phi_{ISC}^{Hd}/\tau_S^{Hd}$), indicating that ISC rate is increased by a factor of 25 because of this heavy atom effect.

From values of k_{ISC}^{H0}, k_{ISC}^{Hd}, τ_S^{H0} and τ_S^{Hd}, the $\eta_{ET,C}^{S}$ in this blend system can be calculated based on following equation by separating rate constant of singlet-singlet ET (k_{ET}^{S}) from other rate constants upon phosphor doping:

$$\eta_{ET,C}^{S} = \frac{k_{ET}^{S}}{\left(\tau_S^{Hd}\right)^{-1}} = \frac{\left(\left(\tau_S^{Hd}\right)^{-1} - k_{ISC}^{Hd} - \left(\left(\tau_S^{H0}\right)^{-1} - k_{ISC}^{H0}\right)\right)}{\left(\tau_S^{Hd}\right)^{-1}} \qquad \text{(S10)}$$

For $SPF_{LMW}/Ir(btp)_2acac$ blend system, k_{ET}^{S} and $\eta_{ET,C}^{S}$ are estimated to be about 2.1×10^{10} s^{-1} and 76%, respectively. The corresponding time scale for singlet-singlet ET (it is inverse of k_{ET}^{S}) is 47 ps. It is much faster than time scale of phosphorescence rise (several hundred ns) shown in Fig. 2a in the main text, supporting the rise component in phosphor transient emission is due to triplet-triplet ET. In the case of $SPF_{HMW}/Ir(btp)_2acac$, $\Phi_{ISC}^{Hd}/\Phi_{ISC}^{H0} = 2.34$ when the η_{ET}^{T} and C_T^{Hd}/C_T^{H0} of 55% and 1.05 are substituted into Equation S9. Since τ_S^{Hd} and τ_S^{H0} are 48 ps and 358 ps for phosphor doped and pure SPF_{HMW}, respectively, along with ISC rates of 1.95×10^{9} s^{-1} (k_{ISC}^{Hd}) and 1.11×10^{8} s^{-1} (k_{ISC}^{H0}) for doped and pure SPF_{HWM}, therefore, k_{ET}^{S} and $\eta_{ET,C}^{S}$ of approximately 1.62×10^{10} s^{-1} and 78 % can be obtained in this blend system based on similar calculation.

Supplementary Note 4. [S]/[T] in the SPF. Supplementary Table 1 is fitting results of Equation 1 to transient emissions of $Ir(btp)_2acac$ in $SPF_{LMW}/Ir(btp)_2acac$ blend system at different applied voltages. We can see that the value of A/C increases with the applied voltage, indicating that large fraction SE can be expected at high applied voltage. Given calculations of $\eta_{ET,C}^{S}$ and η_{ET}^{T}, the [S]/[T] and corresponding χ_S values are shown in the Supplementary Table 1, those for SPF_{HMW} are provided in the Supplementary Table 2.

Supplementary References

1. Tang, K. C., Liu, K. L. & Chen, I. C. Rapid intersystem crossing in highly phosphorescent iridium complexes. *Chem. Phys. Lett.* **386**, 437-441 (2004).
2. Virgili, T. et al. Understanding fundamental processes in poly(9,9-dioctylfluorene) light-emitting diodes via ultrafast electric-field-assisted pump-probe spectroscopy. *Phys. Rev. Lett.* **90**, 247402 (2003)
3. Baldo, M. A. & Forrest, S. R. Transient analysis of organic electrophosphorescence: I. Transient analysis of triplet energy transfer. *Phys. Rev. B* **62**, 10958-10966 (2000).
4. Bernard Valeur. Molecular fluorescence: principles and applications 247-272 (Wiley-VCH, 2001).
5. Rothe, C., King, S. & Monkman, A. Long-range resonantly enhanced triplet formation in luminescent polymers doped with iridium complexes. *Nat. Mater.* **5**, 463-466 (2006).
6. Epshtein, O., Nakhmanovich, G., Eichen, Y. & Ehrenfreund, E. Dispersive dynamics of photoexcitations in conjugated polymers measured by photomodulation spectroscopy. *Phys. Rev. B* **63**, 125206 (2001).
